# In silico high-throughput screening system for AKT1 activators with therapeutic applications in sepsis acute lung injury

**DOI:** 10.3389/fcimb.2022.1050497

**Published:** 2022-12-12

**Authors:** Ziyi Wang, Xuesong Wang, Zhe Guo, Haiyan Liao, Yan Chai, Ziwen Wang, Zhong Wang

**Affiliations:** ^1^ School of Clinical Medicine, Tsinghua University, Beijing, China; ^2^ Department of Liver Intensive Care Unit, Beijing Tsinghua Changguang Hospital, Beijing, China; ^3^ Emergency Department, Beijing Friendship Hospital Affiliated Capital Medical University, Beijing, China

**Keywords:** sepsis, lung injury, HTS - high throughput satellite, apoptosis, AKT1

## Abstract

**Purpose:**

AKT1 is an important target in sepsis acute lung injury (SALI). The current study was aim to construct a high-throughput screening (HTS) system based on the ChemDiv database (https://www.chemdiv.com/complete-list/) and use the system to screen for AKT1 activation agents, which may provide clues for the research and development of new drugs to treat SALI.

**Methods:**

Based on the existing X-ray structure of AKT1 and known AKT activators, a large-scale virtual HTS was performed on the ChemDiv database of small molecules by the cascade docking method and demonstrated both accuracy and screening efficiency. Molecular docking and molecular dynamics simulations were used to assess the stability and binding characteristics of the identified small-molecule compounds. The protective effect of the new highly selective compound on SALI were verified both *in vitro* and *in vivo* experiments.

**Results:**

The small-molecule compound 7460-0250 was screened out as a specific activator of AKT1. Molecular validation experiments confirmed that compound 7460-0250 specifically promoted the phosphorylation of AKT1 and down-regulated the LPS-induced apoptosis of human umbilical vein endothelial cells (HUVECs) by activating the AKT-mTOR pathway. Up-regulated mTOR was detected to directly interact with Bax to reduce apoptosis. *In vivo*, compound 7460-0250 could improved survival rate and alleviated lung injury of sepsis mice induced by cecum ligation and puncture (CLP), parallel with the activation of the AKT-mTOR pathway.

**Conclusion:**

Small-molecule compound 7460-0250 was successfully screened and confirmed as a highly selective AKT1 activator, which is a critical target in the development of new therapeutics for SALI.

## Introduction

Sepsis is defined as life-threatening organ dysfunction caused by dysregulated immune response to infection (Singer et al., 2016). Acute lung injury (ALI) is a common and serious complication of sepsis. The incidence of ALI caused by sepsis is 25%-45%, and the mortality is 50%-60% (Kumar, 2020). Changes in vascular function resulting from vascular endothelial cell injury play an important role in the pathogenesis of SALI. In the pathological state, pathogens cause vascular endothelial injury, vascular endothelial inflammation, and vascular leakage, leading to the entry of immune cells and inflammatory factors aggravating SALI (Hussman, 2020; Pan et al., 2020).

The AKT pathway is closely related to cell apoptosis and has been extensively studied in tumors. AKT inhibitors are used to promote the apoptosis of tumor cells to treat cancer (Hua et al., 2021). In recent years, many studies in the field of sepsis have highlighted that activation of the AKT pathway could inhibit the apoptosis of functional cells, reduce the level of inflammation and oxidative stress, and subsequently improve organ function damage in sepsis (Cao et al., 2021; Li et al., 2022). There are 3 isoforms of AKT—AKT1, AKT2, and AKT3—among which AKT1 is most closely connected to apoptosis (Green et al., 2013; Gong et al., 2017; Kim et al., 2020). Previous studies have confirmed that pyrotoxin could improve apoptosis of LPS-induced HUVECs by promoting AKT1 phosphorylation (Wang et al., 2022). Currently, the only activator targeting AKT is SC79, which binds to the pleckstrin homology (PH) domain of AKT at residue 25 (arginine) (Jo et al., 2012). Based on the existing pharmacophore model and X-ray structure of SC79 (PDB ID: 1UNR), HTS of the small molecule ChemDiv database was conducted in the current study and the highly selective AKT1 activator small molecule compound 7460-0250 was obtained. This novel compound was subsequently characterized through molecular docking and molecular dynamics simulation and *in vitro* with LPS-induced HUVECs. Findings from this study provided a new mean for identifying AKT1 activators that have potential clinical applications in the treatment of SALI.

## Materials and methods

### Regents

Small molecule compound 7460-0250 and MK-2206 were bought from Taosu Bio LTD. (China). Anti-AKT1 (phospho S473) antibody and anti-AKT2 (phospho S474) antibody were bought from Abcam (USA). Anti-Bax antibody and anti-mTOR antibody were from CST (USA). Anti-AKT3 (phospho S472) antibody was from Abnova (China). Annexin V -FITC Apoptosis Kit was purchased from solarbio (China). 10% fetalbovine serum and crystal violet were from Beyotime (China). Cell counting kit-8 was from Beyotime (China).

### Cell culture

Human Umbilical Vein Endothelial Cells (HUVECs) cultivated by Laboratory of Tsinghua Changgung Hospital were cultured in an Incubator (SANYO, Japan) under standard Conditions (37°C, 5% CO2). The experiments were performed after two passages. Cells were cultured in Dulbecco’s ModifIed Eagle Medium (DMEM), high glucose (Gibco, United States) containing.

### Animals

Wild type (WT) male C57BL/6J mice aged 6–8 weeks (Beijing Hufukang Biotechnology Co., LTD, China) were fed under a specifc pathogen-free environment in Tsinghua University. The mice were equally divided into 4 groups (n = 7 mice/group): sham, activator group, SALI, and SALI+activator groups. The surgical procedure was performed as follows: male mice were fasted for at least 12 h and then anesthetized by intraperitoneal injection of tribromoethanol (10 mg/kg). For the sepsis and the activator intervention groups, the cecum was exposed after mid-line laparotomy and ligated immediately below the ileo-cecal valve without causing intestinal obstruction. After being punctured twice with an 18G needle, the cecum was placed back in the peritoneal cavity, and the abdominal wall was closed in two layers. SALI+activator group and activator group were administered with the small molecular compound 7460-0250 (25 and 100 mg/kg) by intraperitoneal injection at 2 h before the CLP operation. For the sham group, the cecum was exposed, and then the abdominal wall was closed in two layers. All the three groups were treated with normal saline just after operation to mimic clinical therapy. At 6 h after the operation, six mice from each group were sacrificed, and inferior lobe of right lung was used as the study sample. And the survival of these four groups at 7 days was recorded. All animal experiments were conducted under the rules approved by the Ethics Committee of Beijing Tsinghua Changung Hospital (protocol code NCT05095324).

### Drug

Small molecule compound 7460-0250 and MK-2206 were obtained from Shanghai Topscience Limited Corporation (Shanghai, China). The powder was stored at −20°C and was dissolved in DMSO adjusted to pH 4.5 with 1 N acetic acid for *in vitro* studies before use.

### Methods

#### Schrodinger

Schrodinger software was used to preprocess the protein to generate the docker grid file, and construct the pharmacophore model of the small molecule compound database and the pharmacophore model of AKT agonist SC79. The pharmacophore model of SC79 was used to match the ChemDiv pharmacophore database model. In addition, ADMET screening high-throughput screening (HTVS) standard precision screening (SP) ultra-high precision screening (XP) was performed, and MMGBSA was used to score and sort, and the top five binding energies were selected for binding mode analysis. The detailed steps and results are shown in the Results section.

### Molecular dynamics simulation

The docking results were selected as the initial structure, Gromacs was used as the dynamics simulation software, AMBER14SB was selected as the protein position, and Gaff2 was selected as the small molecule position. TIP3P water model was used to add solvent to the protein-ligand system to establish the water box, and sodium ion equilibrium system was added. The PME handles electrostatic interactions under elastic simulations using Verlet and CG algorithms, respectively, and minimizes energy for a maximum number of steps (50,000) using the steepest descent method. The Coulomb force cutoff distance and Van der Waals radius cutoff distance are both 1.4 nm. Finally, the canonical system (NVT) and isothermal isobaric system (NPT) equilibrium systems are used, and then 100ns MD simulation is carried out at room temperature and pressure.

### CCK-8

HUVECs were cultured in 96-well plates to 80% confluence, then incubated with the new compound for indicated hours. Cell viability was detected with CCK-8 according to the manufacturer’s instruction. Briefly, after new compound treatment, cells were incubated with 10 mL CCK-8 solution at 37 °C for 2 h and were measured the absorbance of each well at 450 nm.

### Flow cytometry analysis

Cell apoptosis was tested by flow cytometry using an annexin V–FITC apoptosis detection kit. HUVECs were washed twice with phosphate-buffered saline (PBS) and resuspended in 100 µl of1× binding buffer mixed with 5 µl of annexin-V–FITC and 2.5 µl of 7-AAD staining solution for 15 min in the dark at room temperature. After 15 min of incubation, an additional 400 µl of binding buffer was added, and then the cells were analyzed using a flow cytometer (BD, USA). The production of ROS was tested by flow cytometry using an DCFH-DA probe. HUVECs were washed twice with PBS and resuspended in 100 µl PBS mixed with 10µM DCFH-DA for 30 min in the dark at room temperature. Then, the cells were washed with PBS three times, and 300 µl PBS was added before analysis by flow cytometry. 7- AAD and annexin-V assay Q2 + Q3 were used to perform the apoptosis rate (Kepp et al., 2011).

### Western blot

Apoptosis-mediated proteins were analyzed by western blot. The total protein was extracted by radio-immunoprecipitation assay and phenylmethane sulfonylfluoride following the standard protocols for extracting protein. The protein concentrations were quantified using the BCA Protein Assay kit. Samples were electrophoresed in 10 or 8% SDS-PAGE gel and transferred onto a polyvinylidene fluoride membrane. Then, the membrane was blocked in 5% dried milk at 4°C overnight. The membrane was washed with Tris-buffered saline Tween-20 (TBST) three times, followed by incubation with a secondary antibody at room temperature for 40 min. After washing with TBST again, to observe protein signals, substrate luminol reagent and HRP substrate solution were added onto the membrane, 1 ml/membrane, and membrane signals were revealed by an enhanced chemiluminescence immunoblot detection system. The staining intensity of the bands was quantitated by densitometry through ImageJ software. The antibodies used in our study are described above in the “Reagents”. Protein expression levels were defined as gray value, standardized to the housekeeping gene GAPDH, and expressed as a fold of control. All experiments were performed in triplicate and three times independently.

### Immunoprecipitation

For pre-clearance of G protein sugar beads, a total of 25μl of G protein sugar beads (GE Healthcare, Mississauga, ON, Canada) were incubated with primary antibody for 50 min at 4°C. The beads were then mixed with protein from 500μg of cell lysate and spun overnight at 4° C on a spinner. The following day, the beads were centrifuged at 10000 g for 5 min and the supernatant was discarded. The plates were washed 3 times with 1X PBS, mixed with 25μl of 2X SDS buffer, and then boiled at 95°C. The SDS-PAGE and western blot protocols were then followed as mentioned above.

### Hematoxylin-eosin (HE) staining

The lung tissues fixed with paraformaldehyde were embedded in paraffin and sectioned (thickness 5 μm) for HE staining. The pathological changes of lung tissue were observed under the light microscope at 200 x, and according to the pulmonary interstitial edema, alveolar hemorrhage, medium Lung histopathological injury score (0 = normal, 1 = mild, 2 was classified as moderate, 3 as severe, and 4 as extremely severe), and the total score was calculated.

## Results

### The existing AKT activator SC79 was used to screen small molecule compounds

#### AKT1 protein pretreatment

**Figure 1:**
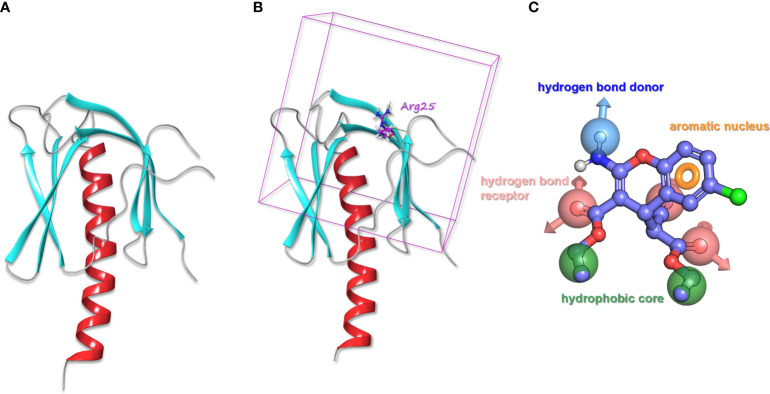
**(A)** The processed structure of AKT1 protein. **(B)** AKT1 protein with the docking box. **(C)** The pharmacophore model of SC79.

Schrodinger’s Protein Preparation module was used for AKT1 Protein (PDB ID Code: 1UNR) pretreatment, that is, water molecules and excess ions were deleted, missing side chains and loop regions were completed, and energy minimization was achieved (**Figure 1A**). Studies have shown that AKT1 activator SC79 binds to the PH region of AKT through interaction with residue R25. According to the sites reported in literature, Arg25 of AKT1 protein is set as the docking center, and the distance 20A around Arg25 is taken as the docking box (**Figure 1B**). Schrodinger’s Glide Grid module is used to generate the docking Grid file. Use of Schrodinger LigPrep module of small molecule ChemDiv database (https://www.chemdiv.com/complete-list/) protons, desalination, hydrogenation, generate tautomer, pretreatment of generating three-dimensional conformation and energy minimization.

#### Construction of pharmacophore model of small molecule compound database

Schrodinger’s Create Phase Database module was used to preprocess tautomers, ionization, etc. The original ChemDiv Database contained 2091084 compounds, and after preprocessing, it contained 2465124 compounds. ChemDiv pharmacophore database model was constructed by using each molecule of ChemDiv database to generate pharmacophore model. There were 120988286 pharmacophore models in total (49.08 pharmacophore models per molecule on average).

#### Construction of AKT agonist SC79 pharmacophore model

According to the molecular structure of AKT activator SC79, the above pretreatment was performed and the pharmacophore model was generated (**Figure 1C**). The pharmacophore model was generated as a total of 7 elements, 1 aromatic ring pharmacophore, 3 hydrogen bond acceptor pharmacophore, 1 hydrogen bond donor pharmacophore, and 2 hydrophobic center pharmacophore.

#### Screening effective pharmacophore

The ChemDiv pharmacophore database model was matched with the pharmacophore model of SC79 in Schrodinger’s Phase Ligand Screening module. A total of 250789 molecules met the criteria of at least six elements of the pharmacophore model and would be screened for the next round of molecular docking. Schrodinger’s QickProp module was used for ADMET screening. A total of 237609 molecules that met the five rules of drug-like properties and did not contain reactivity fragments were retained for the next round of docking screening. Because the last round of pharmacophore screening molecules meet the pharmacophore of positive drug SC79, so the last round of pharmacophore screening 237609 molecules could all docking into the cavity of AKT1, using Schrodinger Glide module high throughput screening (HTVS), The top 10% of the scoring 23761 molecules were selected for the next round of precision screening (SP) docking screening. Schrodinger’s Glide module standard was used for precision screening SP, and the top 10% (2376 molecules) were selected according to the score for the next round of ultra-precision (XP) screening. Schrodinger’s Glide module was used for XP screening, and the top 10% (237 molecules) were scored according to the score for MMGBSA. The top five scores were selected for binding mode analysis (**Table 1**), and molecular dynamics simulation was performed on them.

**Table 1:** MMGBSA scores for selected compounds.

Hits	Title	ID number	Smile	MMGBSAdG Bind(kcal/mol)
**Compound1**	2-(2,4-dioxo-1,3-thiazolidin-5-yl)-N-(3-{[(2,4-dioxo-1,3-thiazolidin-5-yl)acetyl]amino}phenyl)acetamide	7460-0250	c1c(NC(CC2C(NC(=O)S2)=O)=O)cc(NC(CC2C(NC(S2)=O)=O)=O)cc1	-67.46
**Compound2**	diethyl 5-({[(4-(2,3-dimethylphenyl)-5-{[(2-methoxybenzoyl)amino]methyl}-4H-1,2,4-triazol-3-yl)thio]acetyl}amino)-3-methylthiophene-2,4-dicarboxylate	K403-0776	c1cc(c(C(NCc2[nH0](c3c(c(ccc3)C)C)c(SCC(NC3=C(C(C)=C(C(=O)OCC)S3)C(=O)OCC)=O)[nH0][nH0]2)=O)cc1)OC	-62.71
**Compound3**	benzyl {[3-(6-{[2-(3,4-dimethoxyphenyl)ethyl]amino}-6-oxohexyl)-4-oxo-3,4-dihydrothieno[3,2-d]pyrimidin-2-yl]thio}acetate	K292-1366	c1ccc(COC(CSc2[nH0](CCCCCC(=O)NCCc3cc(c(cc3)OC)OC)c(c3c([nH0]2)C=CS3)=O)=O)cc1	-62.54
**Compound4**	N-(3-Chloro-phenyl)-4-{3’-[3-(3-chloro-phenylcarbamoyl)-propyl]-4,4’-dioxo-2,2’-	2159-2999	c1c(cc(NC(CCCN2C(SC(C2=O)=C2C(N(C(=S)S2)CCCC(Nc2cc(Cl)ccc2)=O)=O)=S)=O)cc1)Cl	-61.13
**Compound5**	4-{2-[(1,3-benzodioxol-5-ylmethyl)amino]-2-oxoethyl}-N,N-diethyl-3-oxo-3,4-dihydro-2H-1,4-benzoxazine-6-carboxamide	F118-0638	CCN(C(c1ccc2c(N(CC(NCc3cc4c(cc3)OCO4)=O)C(CO2)=O)c1)=O)CC	-60.12

#### Docking results of small molecule compounds with AKT1 protein molecules

**Figure 2:**
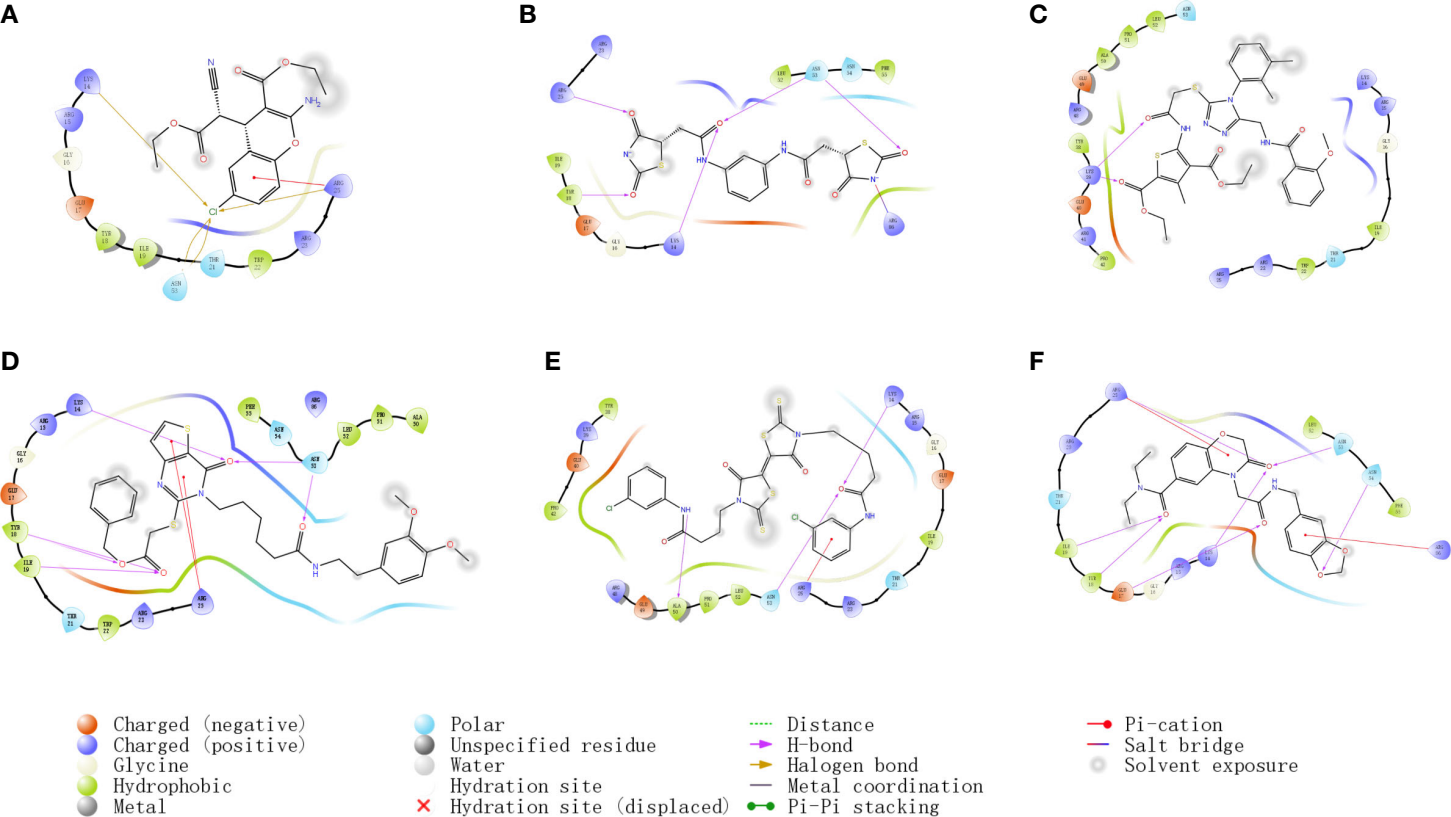
**(A–F)** Docking results of small molecule compounds with AKT1 protein molecules.

In order to compare with the positive compounds, we used the same parameter settings, first performed XP docking on SC79, and calculated the MMGBS binding free energy of SC79: MMGBSA score: -63.21kcal/mol. The results were consistent with those reported in the literature, in which ARG25 was a key amino acid and formed a halogen bond interaction with SC79 and N-PI interaction (**Figure 2A**). Compound 1 binds to the corresponding binding site reported in the literature and forms hydrogen bond interactions with ARG25, a key amino acid reported in the literature. In addition, it can also form hydrogen bond interactions with LYS14, TYR18 and ASN53, and form a salt bridge with ARG86 (**Figure 2B**). Compound 2 forms hydrogen bond interactions with LYS14, TYR18, ILE19 and ASN53 of ATK1, and can also form N-PI interactions with ARG25 (**Figure 2C**). Compound 3 forms hydrogen bond interactions with LYS14, TYR18, ILE19 and ASN53 of ATK1, and can also form N-PI interactions with ARG25 (**Figure 2D**). Compound 4 forms hydrogen bond interactions with LYS14, ALA50, and ASN53 of ATK1, and can also form N-PI interactions with ARG25 (**Figure 2E**). Compound 5 and ARG25 of ATK1 can form hydrogen bond interaction on the one hand and N-PI interaction on the other hand. In addition, it can also form hydrogen bond interaction with LYS14, GLU17, TYR18, ILE19, ASN53 and ASN54. N-pi interaction is formed with ARG86 (**Figure 2F**). The interaction modes and hydrogen bonds between each small molecule compound and AKT1 protein are shown in **Table 2**.

**Table 2:** Interaction of compounds with AKT1.

Compound	IDNUMBER	MMGBSA dG Bind	Interaction				Interacting amino acids
			Hydrogen bonding	n-Pi Interaction	Halogen bond	Salt bridge	
Compound1	7460-0250	-67.46	**4**	**0**	**0**	**1**	LYS14、TYR18、ARG25、ASN53、ARG86
Compound2	K403-0776	-62.71	**2**	**0**	**0**	**0**	LYS39
Compound3	K292-1366	-62.54	**6**	**2**	**0**	**0**	LYS14、TYR18、ILE19、ARG25、ASN53
Compound4	2159-2999	-61.13	**3**	**1**	**0**	**0**	LYS14、ARG25、ALA50、ASN53
Compound5	F118-0638	-60.12	**7**	**2**	**0**	**0**	LYS14、GLU17、TYR18、ILE19、ARG25、ASN53、ASN54、ARG86

Bold values means the predicted bonding bond between the compound and the AKT1 protein.

#### Molecular dynamics simulation results of small molecule compound 7460-0250 and AKT1 protein

**Figure 3:**
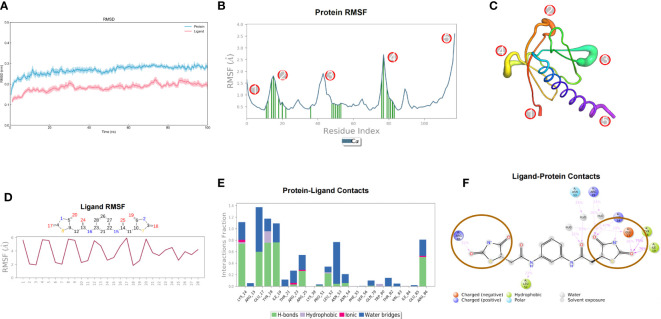
Molecular dynamics simulation results of small molecule compound 7460-0250 and AKT1 protein. **(A)** RMSD; **(B, C)** Protein RMSF; **(D)** Ligand RMSF; **(E, F)** The connection between ligand and protein.

After 100ns molecular dynamics simulation of small molecule compound 7460-0250 and AKT1 protein, trajectory analysis was carried out. Firstly, RMSD of trajectory protein and small molecule were extracted. As shown in **Figure 3A**, protein and small molecule were in a mutually stable state after 10ns. So we could do the following analysis for trajectories from 10 to 100ns. The RMSF of track proteins and small molecules was extracted, as shown in **Figures 3B, C**. There were five places with large RMSF values, namely N segment, C segment and the middle three loop regions. For example, in the RMSF diagram of small molecules (**Figure 3D**), all amino acids of small molecules are basically in a stable state. The interaction mode of the stability interval (10-100ns) of the kinetic trajectory was analyzed, as shown in **Figure 3E**. Amino acids that play important roles in small molecule binding include Lys14, Glu17, Tyr18, Ile19, Arg25, Asn53 and Arg86, whose main roles are hydrogen bonding, water bridge and hydrophobic interaction. The occupancy of interactions formed by 10-100ns (number of frames forming interactions/total number of frames) was counted. As shown in **Figure 3F**, binding of small molecules to proteins mainly depends on 2, 4-thiazolidinediones at both ends.

### Small molecule compound 7460-0250 could specifically activate AKT1 phosphorylation

**Figure 4:**
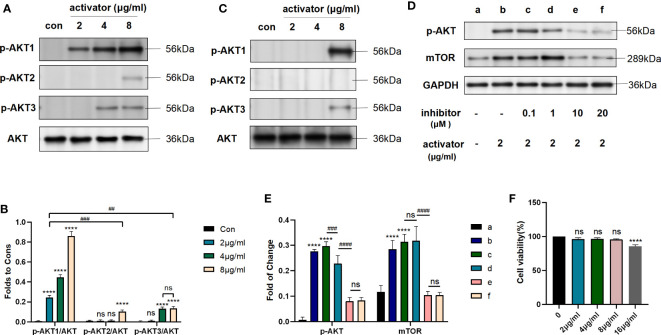
Small molecule compound 7460-0250 could specifically activate AKT1 phosphorylation. **(A, B)** The protein expression of AKT phosphorylation and AKT in HUVECs. **(C)** The protein expression of AKT phosphorylation and AKT in Raw264.7 cells. **(D, E)** AKT inhibitor could inhibit the AKT mTOR pathway activated by the new selectively compound **(F)** Effects of MK2206 on cell viability. Data are presented as mean ± SD (n = 3 per group) of the representative data from three independent experiments; P^##^<0.01, P^####^<0.001, P****<0.001. The asterisk (*) represents the group is statistically different from the Con group. ns, no significant difference.

We used different concentrations of small molecule compound 7460-0250 to induce HUVECs for 8h. As shown in **Figures 4A, B**, with the increase of the concentration of small molecule compound 7460-0250, the p-AKT1/AKT ratio gradually increased in a dose-dependent manner (P < 0.001). The expression of p-AKT2/AKT was significantly increased only when the concentration of small molecule compound 7460-0250 was 8μg/ml (P > 0.05). When the concentration of 7460-0250 was 2 μg/ml and 4μg/ml, there was no significant difference in the expression of p-AKT2/AKT compared with control group (P > 0.05). The p-AKT3/AKT ratio increased slightly when the induction concentration of 7460-0250 was 4 μg/ml and 8μg/ml, which was critically lower than the expression of p-AKT1/AKT at the lowest induction concentration of 7460-0250 (P < 0.001). There was no significant difference in p-AKT3/AKT ratio between the control group and 2μg/ml 7460-0250 group (P > 0.05).

To further verify the activation effect of the new compound in other cell lines, we used different concentrations of small molecule compound 7460-0250 to induce Raw264.7 cells for 8h. As shown in **Figure 4C**, the p-AKT1/AKT ratio increased significantly when the induction concentration of 7460-0250 was 8 μg/ml. The p-AKT3/AKT ratio enhanced slightly when the induction concentration of 7460-0250 was 8 μg/ml. The p-AKT2/AKT ratio could not be up-regulated in these groups.

To determine whether AKT inhibitor could inhibit the AKT-mTOR pathway activated by the new selectively compound or not, western blot was applied. MK-2206 was used as the AKT inhibitor. As shown in **Figures 4D, E**, compared with control group, the expression level of p-AKT and mTOR were enhanced in activator group, and were decreased by MK-2206 in a dose-dependent manner (p < 0.001).

Meanwhile, to detect the cytotoxicity and effective concentration of the new compound, CCK-8 was employed. As shown in **Figure 4F**, the cytotoxicity of the new highly selective compound was also measured to identify an effective and safe dose. The new highly selective compound at 2μg/ml, 4μg/ml, and 8μg/ml had no influence on cell proliferation(p > 0.05). However, the compound at 16μg/ml could reduced the cell proliferation (p <0.001).

### Small molecule compound 7460-0250 could activate AKT-mTOR pathway and down-regulate Bax protein expression

**Figure 5:**
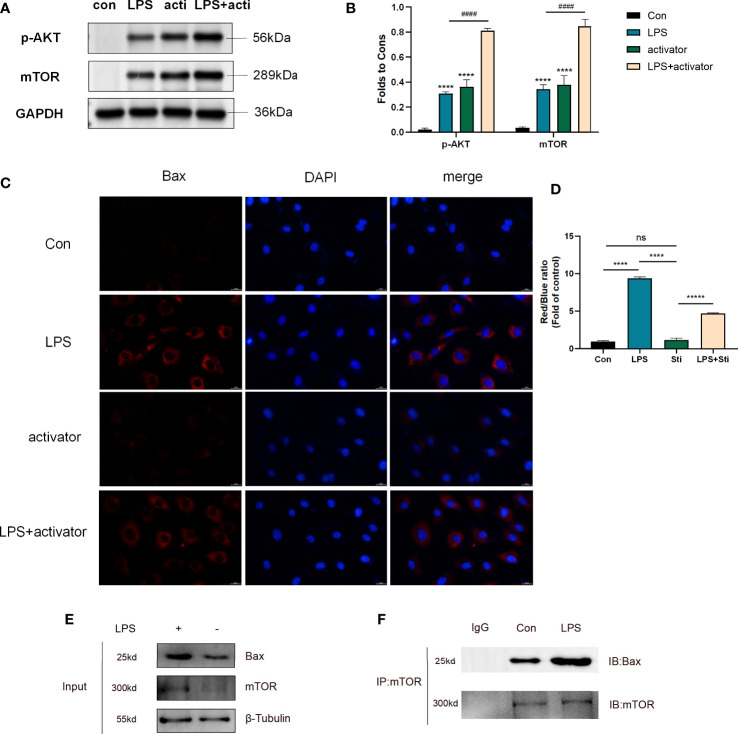
**(A, C)** The protein expression of p-AKT and mTOR in control group, LPS group, activator group, and LPS+activator group. **(B, D)** The protein expression of Bax in control group, LPS group, activator group, and LPS+activator group. **(E, F)** CoIP of mTOR and Bax in control and LPS group. Data are presented as mean ± SD (n = 3 per group) of the representative data from three independent experiments; P^####^<0.001, P****<0.001. The asterisk (*) represents the group is statistically different from the Con group. activator group: compound 7460-0250 group; LPS+activator group: LPS+compound 7460-0250 group. ns, no significant difference.

As previously mentioned, stimulation of HUVECs with 1μg/ml LPS for 6h could successfully induce septic cell model. So we divided cells into four groups: control group, LPS group (1μg/ml LPS), activator group (8μg/ml small molecule compound 7460-0250), and LPS (1μg/ml LPS)+activator (8μg/ml small molecule compound 7460-0250) group. 7460-0250 was added 2h in advance. As shown in **Figures 5A, C**, the expression of p-AKT and mTOR increased slightly after stimulation with LPS or small molecule compound 7460-0250. The expressions of p-AKT and mTOR in LPS+small molecule compound 7460-0250 group were significantly higher than those in single molecule stimulation group (P < 0.05). As shown in **Figures 5B, D**, the expression of Bax in LPS+activator group was significantly increased compared with the control group, and the expression of Bax in LPS+activator group was significantly decreased compared with the LPS group (P < 0.05). There was no significant difference in Bax expression in activator group compared with the control group (P >0.05). CoIP was applied to investigate whether mTOR bounds to Bax in HUVECs. The expressions of mTOR and Bax proteins were detected by Input WB (**Figure 5E**). Empty GFP plasmid failed to bind to Bax, whereas GFP-tagged mTOR bound to Bax in HUVECs (**Figure 5F**).

### The small molecule compound 7460-0250 could down-regulate the apoptosis of LPS-induced HUVECs

**Figure 6:**
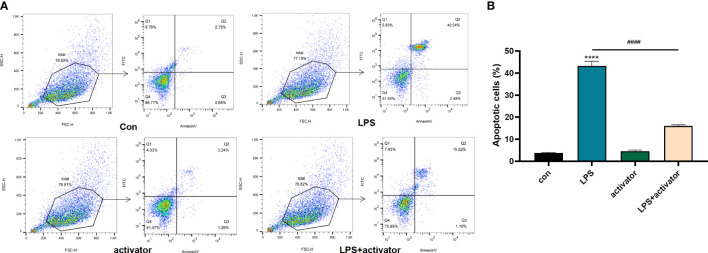
**(A, B)** The flow cytometry results in control group, LPS group, activator group, and LPS+activator group. The small molecule compound 7460-0250 could down-regulate the apoptosis of LPS-induced HUVECs. Data are presented as mean ± SD (n = 3 per group) of the representative data from three independent experiments; P^####^<0.001, P****<0.001. The asterisk (*) represents the group is statistically different from the Con group. activator group: compound 7460-0250 group; LPS+activator group: LPS+compound 7460-0250 group.

As shown in **Figures 6A, B**, the apoptosis rate of HUVECs in LPS group was significantly higher than that of the control group (P < 0.001), and the apoptosis rate of HUVECs in activator group was not statistically different from that of the control group (P > 0.05), while the apoptosis rate in the LPS+activator group was significantly lower (P < 0.001). Apoptosis rates (%) of control group, LPS group, activator group, and LPS+activator group were 3.763 ± 0.281, 43.33 ± 2.068, 4.5 ± 0.51, 15.97 ± 0.65, respectively.

### The small molecule compound 7460-0250 could improve the survival rate of SALI mice induced by CLP

**Figure 7:**
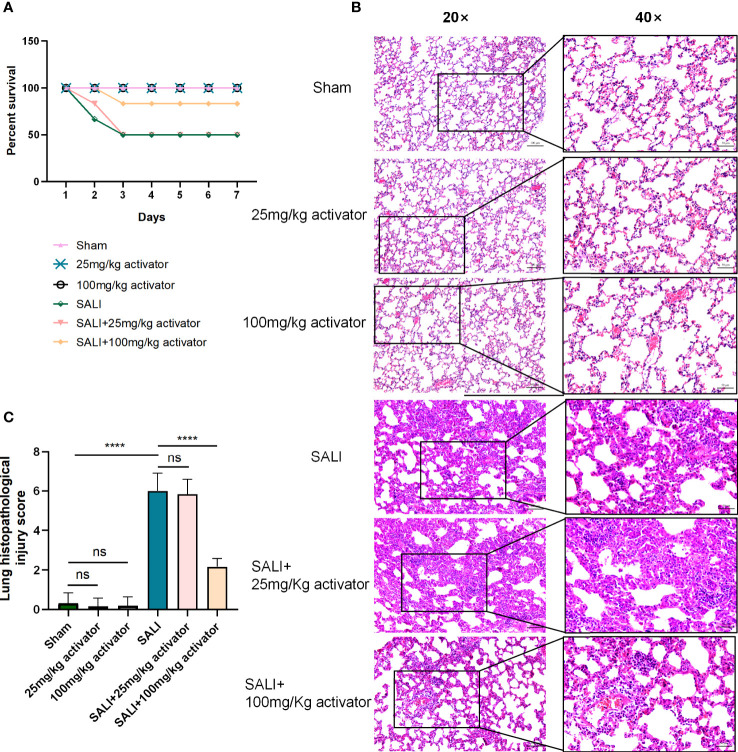
**(A)** The survival rate of sham group, 25mg/kg activator group, 100mg/kg activator group, SALI group, SALI+25mg/kg activator group, and SALI+100mg/kg activator group. **(B, C)** lung issue HE of sham group, 25mg/kg activator group, 100mg/kg activator group, SALI group, SALI+25mg/kg activator group, and SALI+100mg/kg activator group. Data are presented as mean ± SD (n = 3 per group) of the representative data from three independent experiments; Magnification×40, scale bar 50μm. Magnification×20, scale bar 100μm. P****<0.001. 25mg/kg activator group: 25mg/kg compound 7460-0250 group; 100mg/kg activator group: 100mg/kg compound 7460-0250 group; SALI+25mg/kg activator group: SALI+25mg/kg compound 7460-0250 group; SALI+100mg/kg activator group: SALI+100mg/kg compound 7460-0250 group. ns, no significant difference.

Mice were divided into 6 groups: sham group, 25mg/kg activator group, 100mg/kg activator group, SALI group, SALI+25mg/kg activator group, and SALI+100mg/kg activator group. The small molecule compound 7460-0250 was administered by intraperitoneal injection at 2 h before the CLP operation. The 7-day survival rate of the four groups was monitored in our study. As illustrated in **Figure 7A**, while all sham-operated mice and 7460-0250-administrated mice survived to the end of the observation period, half of mice in the SALI group died within 3 days. The survival rate of mice in SALI group were lower than that in the sham group (P < 0.001). The survival rate of mice in the SALI+100mg/kg activator group was essentially improved compared with that in the SALI group (P < 0.001). There was no significant difference in survival rate between SALI+25mg/kg activator group and SALI group (P > 0.05).

### The small molecule compound 7460-0250 could alleviate SALI mice induced by CLP

As shown in **Figures 7B, C**, at 6 h after operation, mice in sham group, 100mg/kg activator group, and 25mg/kg activator group had no obvious congestion, bleeding and inflammatory cell infiltration in lung interstitium. In SALI group, pulmonary interstitial hyperemia, hemorrhage, edema, severe rupture of alveolar capillary wall, and a large number of inflammatory cell infiltration were observed. Mice in SALI+100mg/kg activator group had mild pulmonary interstitial edema, alveolar capillary congestion with a small amount of cleft bleeding, and inflammatory cell infiltration was reduced compared with those in SALI group. Compared with SALI group, SALI+25mg/kg activator group had no significant changes. The lung histopathological injury scores of mice in Sham group, 100mg/kg activator group, 100mg/kg activator group, SALI group, SALI+25mg/kg activator group and SALI+100mg/kg activator group were 0.333 ± 0.21、0.167 ± 0.167、0.2 ± 0.2、6.0 ± 0.365、5.833 ± 0.307、2.167 ± 0.167, respectively. Overall, the difference was statistically significant (P < 0.001). There was no significant difference in the pathological score of lung tissue injury between 25mg/kg Activator group and 100mg/kg Activator group and sham group (P > 0.05), while the pathological score of lung tissue injury in SALI group was significantly higher than that in Sham group (P < 0.001). The pathological injury score of lung tissue in SALI+100mg/kg activator group was significantly lower than that in SALI group (P < 0.001), but there was no significant difference between SALI+25mg/kg activator group and SALI group (P > 0.05).

### The small molecule compound 7460-0250 could activate AKT-mTOR pathway in lung tissue *in vivo*

**Figure 8:**
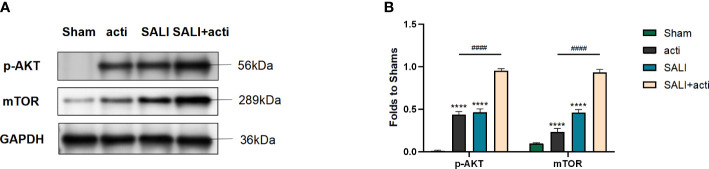
Small molecule compound 7460-0250 could alleviate AKT phosphorylation and mTOR in lung tissue of CLP-induced sepsis mice model **(A, B)** The protein expression of AKT phosphorylation and mTOR in Sham group, activator group, SALI group, and SALI+activator group. Data are presented as mean ± SD (n = 3 per group) of the representative data from three independent experiments. P^####^<0.001, P****<0.001. The asterisk (*) represents the group is statistically different from the Con group. activator group: compound 7460-0250 group; SALI+activator group: SALI+compound 7460-0250 group.

To verify the effect of the new compound *in vivo*, CLP-induced sepsis mice model was employed. As shown in **Figures 8A, B**, compared with sham group, the expressions of p-AKT and mTOR in lung tissue increased slightly in SALI group. Compared with SALI group, the expressions of p-AKT and mTOR in lung tissue in SALI+activator group were significantly higher (P < 0.001).

## Discussion

The systemic inflammatory reaction in sepsis could damage the alveoli, causing alveolar edema and exudation, pulmonary capillary dilatation, congestion, and inflammatory cell infiltration, and could eventually lead to decreased oxygenation capacity of patients, with ALI resulting from progression of the disease (Wang et al., 2019). Although there are numerous predisposing clinical factors for ALI, sepsis is the most common cause, and ALI in patients with sepsis is termed SALI. The main treatments for SALI are anti-infectives, dilatation, diuresis, use of ventilator, and endotracheal intubation. These treatments could save lives and reduce mortality in patients with SALI, but the efficacy is unsatisfactory. Consequently, there is an urgent need to find drugs to prevent and block sepsis (Gotts and Matthay, 2016).

The homeostasis of intercutaneous junctions in alveolar capillary vessels is a key factor for maintaining normal alveolar homeostasis and lung repair after injury (Mehta and Malik, 2006). The alveoli contain abundant pulmonary microvascular endothelial cells, which mediate the transport of molecules from the blood vessels to the lung interstitium. LPS and other activators of lung injury could induce vascular endothelial cell injury (Hou et al., 2019). The most important pathological change of SALI is diffuse alveolar epithelial injury (Mokra, 2020). Under pathological conditions, the destruction of vascular endothelial connections leads to an increase in vascular permeability, the infiltration of inflammatory factors and inflammatory cells, and the production of a cascade reaction to expand the inflammatory effect, resulting in or aggravating SALI (Li et al., 2020). Scholars have found that inducing macrophage polarization from the proinflammatory M1 type to the anti-inflammatory M2 type could reduce ALI caused by sepsis (Jiao et al., 2021). In addition, inhibition of PD-L1 protein expression in neutrophils could promote neutrophil apoptosis and reduce SALI (Wang et al., 2021). These findings demonstrate that the neutrophils and macrophages that exude from the alveolar capillary endothelium then accumulate around alveolar epithelial cells and release numerous inflammatory factors are instrumental in promoting the development of SALI. Therefore maintaining the stability of vascular barrier function and reducing the damage to endothelial cells are vital for the prevention and treatment of SALI.

Activation of the PI3K-AKT-mTOR pathway could alleviate the apoptosis of HUVECs (Lin et al., 2020; Chen et al., 2021). In addition, our previous study revealed that various active components in Reduning, a traditional Chinese medicine, could alleviate LPS-induced apoptosis of HUVECs by targeting AKT1 and activating AKT phosphorylation (Wang et al., 2022). The discovery of AKT dates back to the 1970s, when an oncogene sequence, named AKT8, was identified in murine leukemia viruses, and two homologous oncogenes of AKT8, named AKT1 and AKT2 (also known as PKKB αand PKKB β, respectively), were subsequently identified in human chromosomes (Pastorino et al., 2005). Later, an oncogene called AKT3 (also known as PKBγ) was identified in mammalian cells and was classified as a third isoform of AKT (Gogvadze et al., 2008). Studies of AKT gene subtypes revealed that AKT1 is predominantly involved in apoptosis (Gao et al., 2004), AKT2 is mainly involved in glucose metabolism (Wang et al., 2014), and AKT3 plays a role in the development of the nervous system (Ong et al., 2010).

The existing AKT activator SC79 has the ability to activate all three subtypes of AKT (AKT1, AKT2, and AKT3) (Jo et al., 2012). The current study employed HTS (Kalwat, 2021; Cretin et al., 2021; Tian et al., 2021) to screen out specific activators of AKT1, and more specifically identify those that could protect LPS-induced HUVECs. The small-molecule compound 7460-0250 was identified by HTS of the ChemDiv database using Schrodinger software based on the structure of the existing AKT activator SC79. Compared with the positive control SC79, the novel compound 7460-0250 had a higher MMGBSA score and also formed more hydrogen bond interactions. This indicated that the small-molecule compound 7460-0250 may potentially have improved activity compared with SC79. Therefore, a molecular dynamics simulation was performed on compound 7460-0250 to study the dynamic binding mode of compound 7460-0250 and AKT1, which would provide a theoretical basis for subsequent structural modification. The results of molecular dynamics simulation showed that the small-molecule compound 7460-0250 could stably bind to AKT1 protein, and its binding characteristics were explained.

In molecular biological validation experiments, western blot revealed that small-molecule compound 7460-0250 could specifically activate AKT1 in a dose-dependent manner. Moreover, the protective effect of compound 7460-0250 on LPS-induced HUVECs was subsequently verified. It was found that compound 7460-0250 could downregulate the level of apoptosis-related protein Bax in LPS-induced HUVECs by activating the AKT-mTOR pathway. We therefore investigated the interaction between mTOR and Bax by CoIP. Empty GFP plasmid failed to bind to Bax, whereas GFP-tagged mTOR bound to Bax in HUVECs. Taken together, these data suggest that mTOR interacts with Bax to delay LPS-induced HUVECs apoptosis. Then, we tested the protective effect of compound 7460-0250 in SALI mice induced by CLP. Compound 7460-0250 could up-regulated the survival rate and alleviate pulmonary interstitial hyperemia, hemorrhage, edema, and inflammatory cells infiltration. It was demonstrated that compond 7460-0250 maintained endothelial barrier function by protecting HUVECs from apoptosis, reducing immune cell leakage into the interstitial space and improving further inflammatory responses. This study also demonstrated that HTS is a simple and effective way to explore new therapeutic agents, and our discovery of this novel AKT1 activator may provide a new treatment for sepsis.

## Conclusion

**Figure 9:**
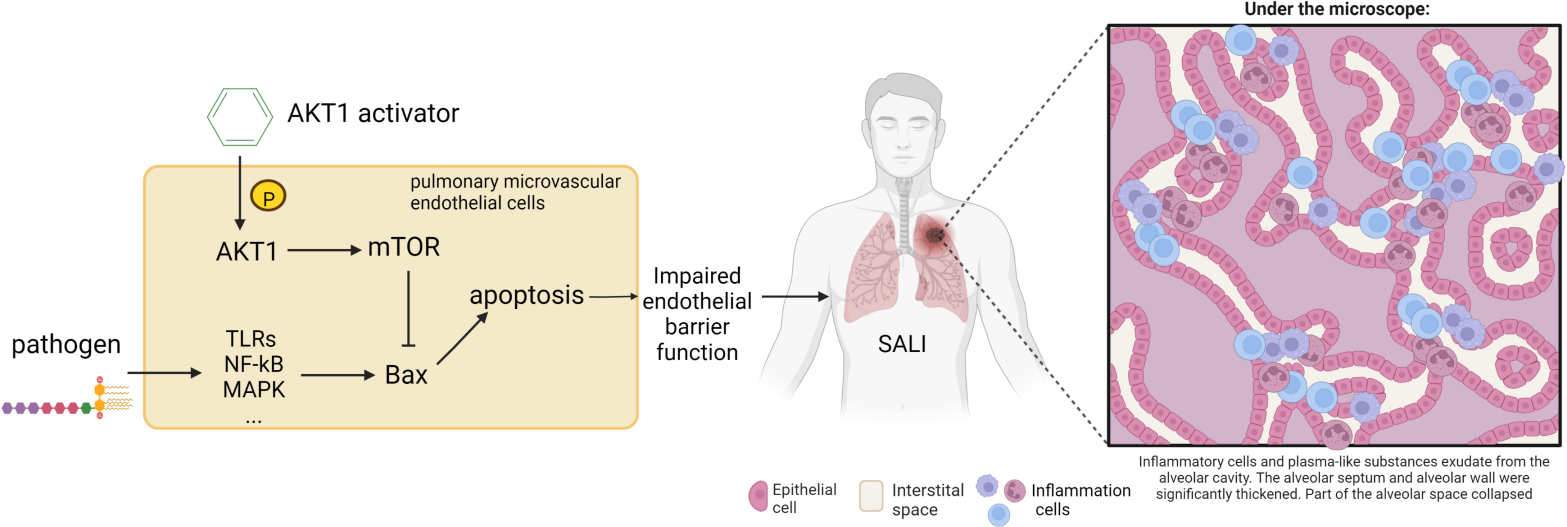
Schematic illustration of the mechanism of the protective effect of AKT1 activator on SALI. The destruction of the endothelial vascular barrier by pathogens leads to the leakage of inflammatory cells into the lung interstitium, resulting in SALI. Compound 7460-0250 may attenuate SALI by activating AKT-mTOR signaling pathway to bind Bax and thereby alleviate alveolar capillary cell apoptosis.

Based on the literature and previous mechanism studies, this study established and optimized a HTS system for activators of AKT1. Through HTS of the ChemDiv database, new highly selective AKT1 activator compound 7460-0250 was identified, and subsequent *in vitro* and *in vivo* experiments confirmed that compound 7460-0250 could attenuate SALI through activating the AKT-mTOR signaling pathway (**Figure 9**). These findings provided a theoretical basis for the research and development of new drugs for sepsis.

### Limitation

As with most studies, the design of the current study is apt to limitations. This study provide a new option for the prevention and treatment of SALI, which has been verified *in vivo* and *in vitro* experiments, but fell short of investigating whether the efficiency of new highly selected small molecule compound is better than SC79 in molecular biological experiments. However, we have demonstrated the advantages of the new highly selective AKT1 activator in molecular docking analysis, so this limitation will not cause a very large bias in the results of the study. We will make further efforts to unearth the role of compound 7460-0250.

## Data availability statement

The original contributions presented in the study are included in the article/Supplementary Material. Further inquiries can be directed to the corresponding author.

## Ethics statement

The animal study was reviewed and approved by Beijing Tsinghua Changgung Hospital (protocol code NCT05095324).

## Author contributions

ZYW conducted to design and writing; XW contributed to cell culture and animal surgery; ZG conducted to western blot; HL contributed to immunofluorescence and flow cytometry; ZWW and YC contributed to HE and results assessment; ZW contributed to supervise this study. All authors contributed to the article and approved the submitted version.
